# Integrated experimental and computational evaluation of atractylenolide III from *Tubipora musica* for anticancer lead identification

**DOI:** 10.1038/s41598-026-68577-5

**Published:** 2026-09-09

**Authors:** Hamada A. A. Noreldeen

**Affiliations:** National Institute of Oceanography and Fisheries, NIOF, Cairo, Egypt

**Keywords:** Atractylenolide III, *Tubipora musica*, Liver Cancer (HepG2), Cytotoxic activity, Computational drug design, Molecular docking, ADMET, Biochemistry, Cancer, Chemistry, Computational biology and bioinformatics, Drug discovery

## Abstract

Hepatocellular carcinoma (HCC) remains a major global health challenge, with over 900,000 new cases annually and a pronounced male predominance. In pursuit of novel, low-toxicity therapeutics, this study isolated Atractylenolide III from the marine coral *Tubipora musica* and demonstrated measurable, dose-dependent cytotoxic activity against HepG2 liver cancer cells, achieving an IC_50_ value of 15.50 µg/mL and reducing cell viability to 25.0% at 25 µg/mL. To investigate potential molecular targets and propose hypothetical mechanism pathways, we employed a multi-layered computational strategy. Molecular docking across 76 cancer-associated proteins identified a strong binding affinity (-9.6 kcal/mol) with CD1b (PDB ID: 1GZP), a T-cell surface glycoprotein key to lipid antigen presentation and tumor immunology. Short-term molecular dynamics (MD) simulations were conducted to provide a preliminary structural-viability filter for this complex under physiological conditions. Pharmacophore modeling expanded the screening to 1,513 structurally related compounds, pinpointing ZINC64701878 as a superior candidate with enhanced binding affinity (-12.7 kcal/mol). Density functional theory (DFT) calculations supported its high reactivity, and pharmacokinetic profiling suggested favorable drug-likeness. Together, these findings propose a theoretical framework for marine-derived compounds as potential leads for HCC therapy. While the cytotoxicity assay provides initial phenotypic support, the primary contribution of this study lies in establishing a predictive computational-experimental framework to guide future in vivo and mechanistic investigations.

**Supplementary Information:**

The online version contains supplementary material available at https://doi.org/10.1038/s41598-026-68577-5.

## Introduction

Liver cancer, specifically hepatocellular carcinoma (HCC), poses a significant global health concern, with over 900,000 new cases diagnosed annually. HCC is not only the most common primary liver malignancy but also ranks as the fourth leading cause of cancer-related mortality worldwide, reflecting both its high incidence and poor prognosis. Its pathogenesis is closely associated with chronic liver diseases such as viral hepatitis, alcohol-related liver injury, and nonalcoholic fatty liver disease, which further amplify its global health impact^1,2^. Notably, 69.8% of HCC cases occur in males, resulting in a male-to-female incidence ratio of 2.66. Consequently, HCC ranks as the fifth most common cancer in men, the ninth in women, and the sixth overall, highlighting the critical need for innovative therapeutic approaches^3^. The HepG2 cell line, a widely recognized in vitro model for HCC, has been instrumental in assessing anticancer compounds due to its well-characterized nature and extensive use in pharmacological and toxicological research since the 1970s^4^. Marine ecosystems, particularly coral reefs, have garnered attention as rich sources of bioactive compounds, as corals produce unique secondary metabolites that enable them to survive in competitive environments. Recent research underscores the potential of marine-derived molecules, which exhibit diverse structural features and potent anticancer properties, including cytotoxic effects, apoptosis induction, and metastasis inhibition. These compounds provide promising scaffolds for drug development, especially in addressing therapy-resistant cancers such as HCC^5^. Given the urgent need for novel and effective anticancer drugs, researchers continue to explore marine organisms as valuable sources of new therapeutic agents.

Corals have gained significant attention for their ability to produce sesquiterpenoids and prostaglandins, which exhibit remarkable cytotoxic properties. Notably, metabolites derived from corals have shown effectiveness against HCC by influencing apoptosis-related pathways^6,7^. Among marine-sourced bioactive compounds, sesquiterpene lactones, such as Atractylenolide III, are particularly noteworthy due to their unique structural elements, including a fused furan ring and α-methylene-γ-lactone moieties. These structural features are closely associated with apoptosis induction through mechanisms such as NF-κB inhibition and nitric oxide (NO) generation^8,9^. Research has demonstrated that Atractylenolide III possesses a diverse pharmacological profile, encompassing both anti-inflammatory and anticancer properties, with notable effects on immune modulation and cancer cell suppression^10–12^. Specifically, Atractylenolide III has demonstrated distinct, target-specific anticancer effects across several cancer models; it induces apoptosis in human lung carcinoma (A549) cells via the mitochondria-mediated pathway, triggers cell cycle arrest and apoptosis in colorectal cancer (HCT-116) cells by regulating the Bax/Bcl-2 signaling pathway, and suppresses breast cancer progression by downregulating JAK3/STAT3-dependent IDO expression^9,12,13^. Also, it exerts tumor-suppressive effects in HCC by upregulating miR-195-5p, which in turn directly targets and downregulates the oncogenic receptor FGFR1. This cascade inhibits tumor cell proliferation and induces apoptosis^14^. These findings reinforce the growing interest in marine-derived natural products as valuable sources of multitarget therapeutic agents capable of addressing drug resistance in cancer treatment.

Modern drug discovery increasingly integrates computational biology and chemistry with experimental workflows to accelerate lead optimization while reducing preclinical translation costs^15,16^. Virtual screening, physicochemical profiling, and ADMET prediction offer rapid, early-stage evaluations of pharmacokinetics and drug-likeness^12^. Furthermore, molecular docking and molecular dynamics (MD) simulations provide critical, atomistic insights into ligand-receptor interactions, binding affinities, and complex stability under simulated physiological conditions^17,18^. When complemented by pharmacophore modeling and density functional theory (DFT) calculations^19,20^, these predictive methods establish a robust framework to systematically prioritize marine-derived natural products, such as atractylenes, for targeted therapeutic development.

**Scheme 1:**
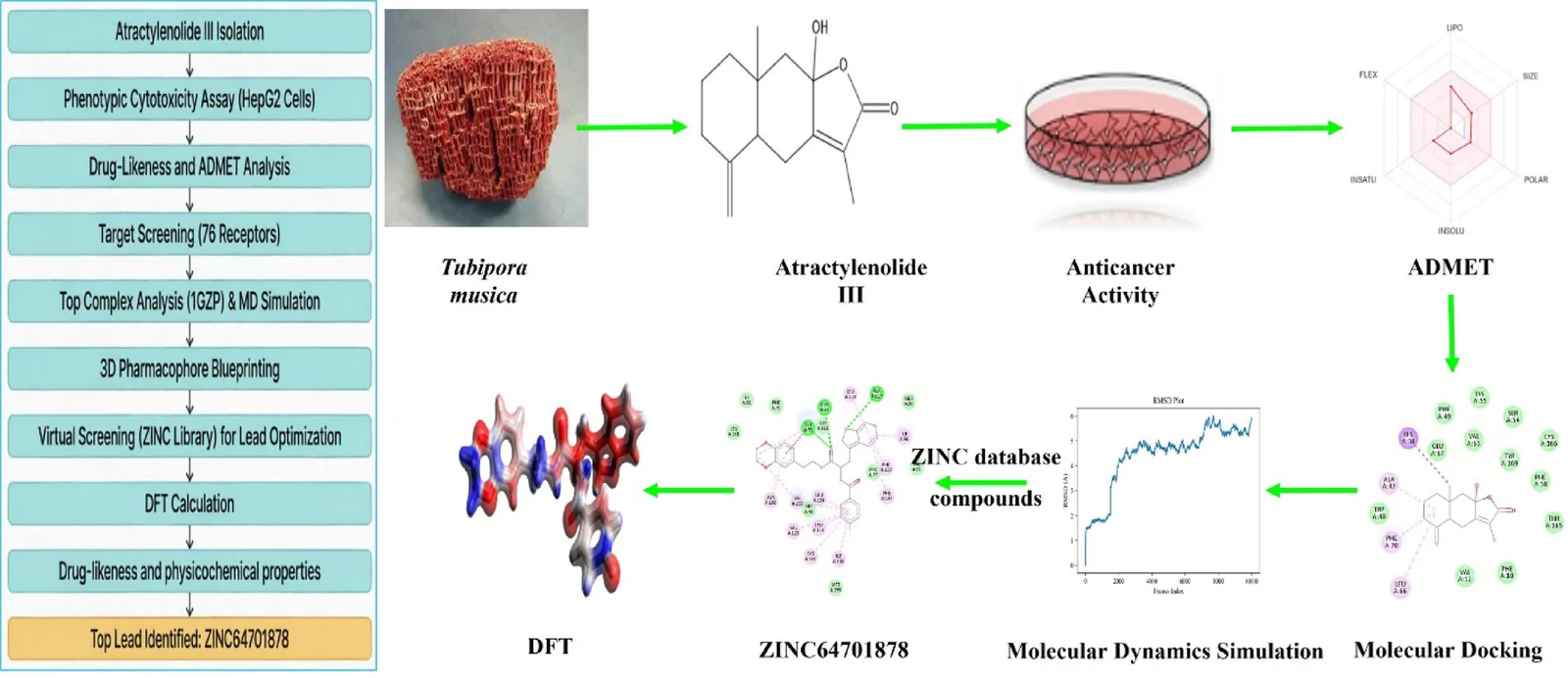
A diagram illustrating the sequential process followed in the study.

This study aimed to assess the cytotoxic effects of Atractylenolide III on HCC and investigate its mechanism of action using a comprehensive computational approach. Initially, the cytotoxic potential of Atractylenolide III against HepG2 cells was evaluated. Subsequently, an integrated computational workflow was applied, beginning with physicochemical property prediction, ADMET profiling, and drug-likeness assessment via SwissADME. To identify potential molecular targets, SwissTargetPrediction^21^ was utilized alongside literature data^8–10,22^, selecting over 70 cancer-associated proteins, including kinases, immune regulators, and apoptosis regulators, for molecular docking. Among these, the lipid antigen-presenting molecule CD1b (PDB ID: 1GZP), which plays a pivotal role in T-cell-mediated tumor immune surveillance^23^, was identified as a primary candidate of interest for downstream structural and dynamic optimization. Therefore, the top ligand-protein complexes, determined through AutoDock Vina scoring^17^, were then subjected to MD simulations to evaluate preliminary structural viability. Additionally, 3D pharmacophore modeling was performed to identify key interaction features for virtual screening, leading to the selection of structurally optimized analogs. The best-performing ligand, ZINC64701878, was identified, and its drug-likeness was confirmed. These findings support the hypothesis that Atractylenolide III exhibits measurable anticancer activity against HepG2 cells, and computational modeling provides insights and guides future drug optimization strategies. Scheme 1 presents a detailed representation of the step-by-step workflow employed in this study.

## Materials and methods

### Isolation and characterization of atractylenolide III

*Tubipora musica* was collected from the coastal waters of the Red Sea, Egypt. The collection was under the official authorization, guidelines, and permits issued by the National Institute of Oceanography and Fisheries (NIOF), Cairo, Egypt, ensuring compliance with local laws and international conservation frameworks. Initial identification was performed through field photography, followed by laboratory confirmation. The frozen hard coral (4 kg) was chopped into small pieces and extracted using 100% methanol at room temperature. The combined methanolic extracts were concentrated to yield a brown gum (10 g). The dried extract was subjected to silica gel column chromatography and eluted with an n-hexane-methanol gradient. Fractions were collected and grouped based on their thin-layer chromatography (TLC) profiles. A selected fraction (1 g), eluted with 100% dichloromethane, was further separated via silica gel column chromatography, resulting in the isolation of Atractylenolide III (8 mg). The compound was dissolved in dichloromethane and purified through silica gel chromatography using a mobile phase of hexane, dichloromethane, and methanol (7:4:0.25). Additional purification was performed using a Sephadex LH-20 column with the same solvent system. For structural characterization, ^1^H and^13^ C NMR spectra were recorded in CDCl₃ using a Varian 300 MHz and Varian Unity INOVA 600 spectrometer (300/600 MHz for^1^ H and 75/125 MHz for^13^ C). Chemical shifts (δ) were reported in ppm relative to tetramethylsilane (TMS) as an internal standard, with coupling constants (J) provided in Hz.

### Cytotoxicity assay

The human cancer cell lines used in this study were obtained from the National Research Center (NRC), Cairo, Egypt. According to institutional and national ethical guidelines, research utilizing established, secondary commercial/repository cell lines does not require dedicated institutional ethical committee approval. The cytotoxic potential of the tested compound was evaluated at the National Cancer Research Center (NRC), Cairo, Egypt, following the Skehan et al. protocol^24^. In summary, HepG2 cells were seeded in a 96-well microplate at a density of 10^4^ cells/well and incubated for 24 h to allow proper adherence. Subsequently, varying concentrations of the compounds were introduced to the monolayer, with each concentration tested in triplicate. The treated cells were incubated for 48 h at 37 °C in a 5% CO_2_ atmosphere. After incubation, the cells were fixed, washed, and stained using Sulforhodamine B (SRB) stain. The excess stain was removed by acetic acid washing, and the bound stain was solubilized using Tris(hydroxymethyl)aminomethane-EDTA buffer. The color intensity, which correlates with cell viability, was measured using an ELISA microplate reader (Sunrise, Bas TEcan, 5082, Grodig, Austria). The relationship between drug concentration and the surviving cell fraction was analyzed to generate dose-response survival curves for each treatment (*n* = 3).

### Drug-likeness and ADMET analysis

Assessing pharmacokinetic properties is crucial for understanding the oral bioavailability and safety of drug candidates. To achieve this, SwissADME (http://www.swissadme.ch/) and pkCSM (https://biosig.lab.uq.edu.au/pkcsm/) were utilized to predict the ADMET (Absorption, Distribution, Metabolism, Excretion, and Toxicity) profiles and other in silico pharmacokinetic parameters of the designed compounds. Additionally, the RDKit Python library was employed to compute supplementary molecular properties and drug-likeness indices, specifically the Ring Count, Quantitative Estimate of Drug-Likeness (QED) score, and general Toxicity Flags^25^. Furthermore, target selection was conducted based on previous literature^8–11^ and computational screening using SwissTargetPrediction (http://www.swisstargetprediction.ch/).

### Protein-ligand pharmacophore (PLP) detection and molecular docking

Molecular docking simulations play a crucial role in predicting the binding orientation of Atractylenolide III with its target protein, providing insights into binding affinities and molecular interactions. The protein structures and 3D ligand structures were obtained from the Protein Data Bank (PDB) (https://www.rcsb.org) and PubChem (https://pubchem.ncbi.nlm.nih.gov/), respectively. To accommodate the structural diversity of the 76 screened protein targets, a two-tiered grid box definition strategy was employed based on the presence or absence of co-crystallized structural ligands.

First, for targets containing co-crystallized structural ligands, the Protein-Ligand Pharmacophores (PLP) method was applied. Here, the active site was determined based precisely on the coordinate centroids of the co-crystallized binding partners to ensure active-site specificity. The active site residues and specific grid box locations (X, Y, Z coordinates and dimensions) were identified and verified using Discovery Studio v21.1.0 (BIOVIA, 2021). Second, for proteins lacking co-crystallized structural ligands, an Automated Grid Box Selection was utilized. In these cases, grid coordinates were established using AutoDock MGLTools v1.5.7^26,27^, leveraging its default pocket-detection settings to construct a grid covering the most probable binding cavity as predicted by discovery algorithms.

To ensure search-space exhaustiveness and reproducibility, docking calculations for the top prioritized complexes were performed in independent quintuplicate runs (*n* = 5) at an exhaustiveness level of 8, with comprehensive coordinates and validation parameters detailed in the Supplementary information. Before docking, protein preparation was carried out by removing water molecules and existing ligands, followed by the addition of polar hydrogen atoms and Kollman charges. The protein and ligand structures (downloaded from PubChem as SDF files) were converted into PDBQT format using AutoDock MGLTools. Finally, flexible docking simulations were executed using AutoDock Vina v1.1.2^28^, enabling the identification of optimal ligand binding conformations.

### Molecular dynamics (MD) simulation

Molecular Dynamics (MD) simulation is a fundamental computational technique used to evaluate the stability of a protein-ligand complex over time within a simulated biological environment. By capturing atomic-scale motions over time, MD provides insights beyond static docking results, including conformational flexibility and interaction persistence, which are essential for evaluating the reliability of predicted binding modes^29–37^. By providing insights into structural fluctuations and conformational changes, MD simulations help determine whether the complex achieves a stable conformation. In this study, MD simulations were conducted to analyze the stability of the top-ranked docking complex, specifically the human T-cell surface glycoprotein CD1b (1GZP)-Atractylenolide III binding complex. The necessary files for MD simulations were generated using Visual Molecular Dynamics (VMD) v1.9.3. First, the coordinates of 1GZP and Atractylenolide III were converted into PDB format, and the protein was parameterized using an automatic PSF builder. The CHARMM-GUI web server (http://www.charmm-gui.org/?doc=input) was utilized for ligand topology generation, applying the CHARMM force field. The protein-ligand complex was solvated using a TIP3P water box, ensuring a 5.0 Å solvent buffer around the system, followed by ionization with NaCl to neutralize the system.

The MD simulation parameters included a 2 fs timestep, Particle Mesh Ewald (PME) method for electrostatics, and Langevin dynamics for temperature regulation at 310 K. Charge calculation methods, such as Restrained Electrostatic Potential (RESP) and Austin Model 1 with Bond Charge Correction (AM1-BCC), were applied within the CHARMM force field to accurately model interactions for both proteins and small molecules. Periodic boundary conditions (PBC) and Maxmin TCL scripting were implemented for system constraints. The system underwent energy minimization for 100 steps, followed by 500,000 simulation steps (1 ns) to monitor the preliminary structural viability. Finally, Root Mean Square Deviation (RMSD) and Root Mean Square Fluctuation (RMSF) analyses were performed to assess the preliminary structural stability of the docked complex throughout the simulation.

### Pharmacophore modeling and virtual screening

The three-dimensional (3D) pharmacophore models utilized in this study were derived from the optimal binding pose of Atractylenolide III obtained through molecular docking. The pharmacophore maps of the top-ranked ligand conformation were constructed using ZINCPharmer (http://zincpharmer.csb.pitt.edu/), which enables compound screening based on 3D spatial features. This approach effectively captures the spatial arrangement of key molecular interactions, such as hydrophobic networks and hydrogen bonding, which are essential for ligand-protein binding affinity. The generated pharmacophore model was structurally validated by confirming that all selected features precisely mapped to key coordinating residues within the active binding pocket of CD1b. Following pharmacophore generation and validation, virtual screening was conducted against the ZINC compound library to identify structurally similar molecules with potential biological activity. The screening constraints against the ZINC database were strictly restricted to structures matching a specific five-feature pharmacophore hypothesis: two hydrophobic regions, one hydrogen bond donor, and two hydrogen bond acceptors. To isolate the most promising hits, multi-tiered selection criteria were applied; compounds were filtered and selected only if they yielded an alignment RMSD fit < 0.35 Å, a molecular weight ≤ 500 g/mol, and ≤ 10 rotatable bonds. The filtered ZINC library hits were subsequently converted into PDBQT format and prepared for docking simulations. Final prioritization of the screened hits was determined by their binding energies calculated via AutoDock Vina. This integrated pharmacophore-based virtual screening strategy facilitates the systematic discovery of novel bioactive candidates that share essential spatial and electronic interaction characteristics with the lead molecule, maximizing the likelihood of identifying drug candidates with improved binding properties.

### Density functional theory (DFT) analysis

Density Functional Theory (DFT) is widely recognized for its accuracy and cost-efficiency in investigating fundamental material properties, including energy landscapes, structural configurations, electrical characteristics, and optical properties^38^. Generally, DFT calculations were performed to analyze crucial quantum chemical descriptors. These calculations provided deeper insights into the electronic behavior, chemical reactivity, and kinetic stability of the investigated molecules^39–41^. In this study, to further explore molecular reactivity, various quantum molecular descriptors were computed, such as Highest Occupied Molecular Orbital (HOMO), Lowest Unoccupied Molecular Orbital (LUMO), band gap energy (ΔE = LUMO - HOMO), chemical hardness (η = (LUMO - HOMO) / 2), chemical softness (S = 1/η), electronegativity (χ = - (HOMO + LUMO) / 2), and electrophilicity index (ω = (χ²) / (2η)).

The molecular geometry was initially optimized using the MMFF94 force field through Avogadro visualization software^42^ before conducting DFT-based optimizations. The ORCA 4.2.1 program^43^ was utilized for geometry optimization and quantum mechanical calculations, employing the Becke-Perdew (BP) density functional method combined with the split-valence double-zeta basis set (def2-SVP). The BP density functional combined with def2-SVP is recognized as a highly cost-effective and structurally reliable level of theory for geometry optimizations and electronic properties of organic drug-like molecules, striking an optimal balance between accuracy and computational cost^44^. Furthermore, a Molecular Electrostatic Potential (MEP) diagram was generated using Avogadro, visually depicting the electrostatic potential (ESP) mapped onto the electron density (ED) surface. The color gradient from deep red to deep blue effectively illustrates the charge distribution across the molecular surface, providing insights into reactive sites and intermolecular interactions^20^.

## Results and discussion

### Characterization of atractylenolide III

The ^1^H NMR analysis of Atractylenolide III exhibited characteristic signals corresponding to two methyl groups at δH 1.01 (H-14) and δH 1.8 (H-13), along with two singlet peaks at δH 4.5 (H-15a) and δH 4.85 (H-15b), indicating the presence of exomethylene protons (Figure S1A and Table S1). Furthermore, ^13^C NMR analysis (Figure S1B and Table S2) identified 15 carbon signals, which were classified using DEPT analysis. These included two methyl groups at δC 8.3 (C-13) and δC 16.6 (C-14), six methylene groups at δC 41.4 (C-1), δC 22.4 (C-2), δC 36.2 (C-3), δC 24.6 (C-6), δC 51.4 (C-9), and δC 106.8 (C-15), a single methine carbon at δC 51.8 (C-5), and six quaternary carbons at δC 148.9 (C-4), δC 160.8 (C-7), δC 103.4 (C-8), δC 36.7 (C-10), δC 122.3 (C-11), and δC 172.0 (C-12). Additionally, the ^13^C NMR spectrum confirmed the presence of an oxygenated quaternary carbon at δC 103.4 (C-8), an exomethylene carbon at δC 106.8 (C-15), three olefinic quaternary carbons at δC 148.9 (C-4), δC 160.8 (C-7), and δC 122.3 (C-11), as well as a keto functional group at δC 172.0 (C-12). These spectral data align with previously reported findings, confirming that the isolated compound is Atractylenolide III, which has also been identified in several medicinal plants, including *Trattinnickia aspera*^45^, *atractylodes macrocephala*^46^, *and Codonopsis pilosula*^47^.

### Cytotoxic activity of atractylenolide III

**Fig. 1:**
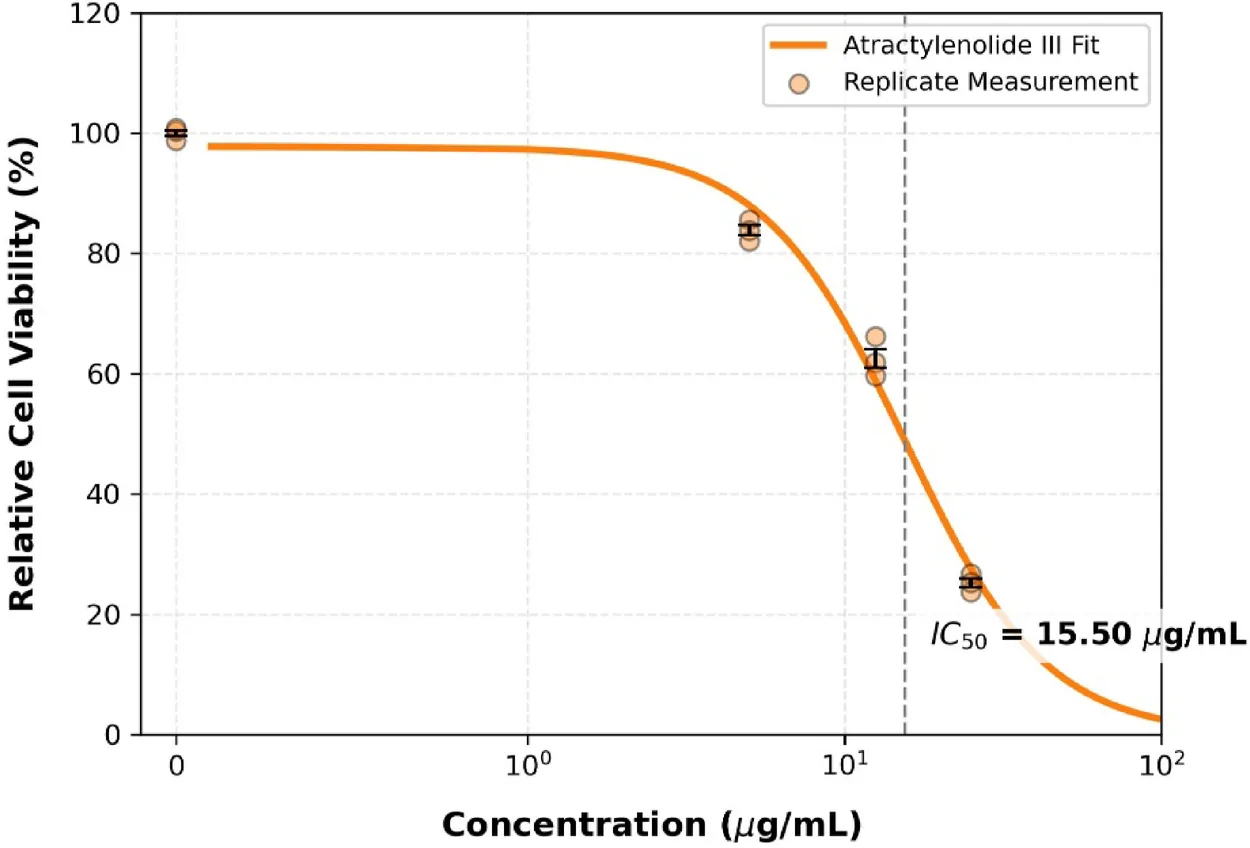
Cytotoxic activity of Atractylenolide III against HepG2 cell lines (*n* = 3 ± SD).

Figure 1 illustrates the measurable cytotoxic effects of Atractylenolide III against hepatocellular carcinoma (HepG2) cells, demonstrating a dose-dependent manner in cell viability. Specifically, cell decline was characterized by viable cell populations of 84%, 62%, and 25%, at treatment concentrations of 5, 12.5, and 25 µg/mL, respectively, compared to the untreated control (100% at 0 µg/mL). Additionally, the calculated IC_50_ value of 15.50 µg/mL suggests a moderate cytotoxic effect (compounds with IC_50_ values > 50 µg/mL are typically considered weakly cytotoxic)^48^. The substantial reduction in viability at higher concentrations suggests that Atractylenolide III may exert its effects through mechanisms such as apoptosis induction or cell cycle arrest, which are commonly associated with sesquiterpene lactones^13,22,49^. These results are consistent with recent studies that have highlighted the anticancer potential of Atractylodes-derived compounds in hepatoma cell lines^50^. However, further investigations are required to identify the potential molecular targets and mechanisms of action. To address this, several computational drug design approaches have been employed in this study to explore potential target interactions and binding affinities.

### Drug-likeness and ADMET analysis

To minimize the likelihood of failure during the preclinical and clinical phases of drug development, in silico analysis of a compound’s ADMET (Absorption, Distribution, Metabolism, Excretion, and Toxicity) properties is employed^51–53^. This approach provides critical insights into a compound’s pharmacokinetics, bioavailability, and potential toxicity when administered orally^54^. Atractylenolide III exhibits favorable physicochemical properties for drug development, with a molecular weight of 248.32 g/mol and a topological polar surface area (TPSA) of 46.53 Å², which are typically associated with good membrane permeability^55^. These findings align with Lipinski’s Rule of Five and Veber’s criteria, as Atractylenolide III does not violate any of these rules, indicating potentially high oral bioavailability^56,57^. According to Lipinski’s Rule, drug-like molecules should have no more than one violation across the following parameters: molecular weight ≤ 500 g/mol, hydrogen bond donors ≤ 5, hydrogen bond acceptors ≤ 10, and LogP ≤ 5 ^56^. Meanwhile, Veber’s criteria suggest that optimal oral bioavailability is associated with TPSA ≤ 140 Å² and ≤ 10 rotatable bonds^57^. Additionally, Atractylenolide III exhibits theoretical structural rigidity and stability, as reflected by the absence of rotatable bonds (0) and a high fraction of sp³-hybridized carbons (0.67) (Table 1). These characteristics may contribute to enhanced metabolic stability and synthetic accessibility, further supporting the compound’s potential as a promising drug candidate^55^.

Lipophilicity is a crucial parameter in drug development, as it significantly impacts ADME properties^58^. The analyzed compound demonstrates moderate lipophilicity, with a consensus LogP value of 2.65, indicating a potential well-balanced hydrophilic-lipophilic profile that could support efficient absorption, distribution, and oral bioavailability^55^. Additionally, the compound exhibits optimal hydrogen-bonding properties (3 hydrogen bond acceptors and 1 donor), which might contribute to balanced membrane permeability and solubility, further enhancing its drug-likeness^55^. Moderate solubility is often advantageous, as it enables the ligand to efficiently travel through systemic circulation upon administration, increasing its therapeutic potential^59^. Further, water solubility is a key determinant of a drug’s absorption and bioavailability. To comprehensively assess solubility, various predictive models (i.e., ESOL^60^, Ali^61^, and SILICOS-IT) were employed (Table 1). The results indicate that the isolated compound theoretically exhibits high aqueous solubility, which is favorable for oral drug administration, as it facilitates enhanced absorption and systemic availability^55^.

From a pharmacokinetic perspective, high gastrointestinal (GI) absorption is a highly desirable trait for oral drug candidates. While Atractylenolide III possesses blood-brain barrier (BBB) permeability, which is not directly required for localized HCC therapy unless addressing secondary brain metastases, this property provides a comprehensive pharmacokinetic profile of the isolated lead scaffold. Further, exhibiting high GI absorption and BBB penetration, with a skin permeation coefficient (Log Kp) of -6.32 cm/s, might indicate low dermal absorption^62^. Additionally, medicinal chemistry profiling highlights that the compound presents no PAINS (Pan-Assay Interference Compounds) alerts, suggesting a low likelihood of false-positive bioactivity^63^. However, one Brenk alert may be related to the lactone moiety, suggesting potential reactivity, which may require structural optimization to reduce off-target interactions^64^. The synthetic accessibility score of 4.21 indicates that the compound can be synthesized in a laboratory setting with moderate ease. Furthermore, drug-likeness analysis confirms that Atractylenolide III meets the criteria of Lipinski, Ghose, Veber, and Muegge rules, reinforcing its viability as a lead compound. Its quantitative estimate of drug-likeness (QED) score of 0.53 suggests moderate drug-likeness, positioning it as a potentially promising candidate compared to approved pharmaceuticals^65^.

Bioavailability refers to the fraction of an administered drug that reaches systemic circulation in its unmodified form and plays a crucial role in determining absorption rate and therapeutic efficacy^66^. Generally, intravenously administered drugs exhibit higher bioavailability compared to oral formulations^54^. This parameter is fundamental in defining appropriate drug dosage and therapeutic effectiveness^54,67^. Atractylenolide III exhibits a bioavailability score of 0.55 (Table 1), indicating moderate oral bioavailability^55^. While this suggests promising potential as an oral drug candidate, further optimization strategies may be required to enhance its pharmacokinetic performance. Approaches such as prodrug formulation or salt modifications could improve bioavailability while maintaining favorable drug-likeness properties. These refinements would enhance its drug-like characteristics, positioning Atractylenolide III as a potential candidate for preclinical drug development.

The calculated physicochemical and pharmacokinetic profiles highlight the therapeutic potential of Atractylenolide III as a liver-targeted agent with high systemic efficiency^14^. Structurally, the compound exhibits a balanced TPSA (46.53 Å²) and LogP (2.65). This structural profile facilitates excellent passive gastrointestinal absorption while maintaining optimal water solubility, effectively preventing premature metabolic clearance, a critical pharmacodynamic bottleneck in liver-targeted drug delivery^10^. Furthermore, the predicted BBB permeability index (0.251) suggests low-to-moderate central nervous system penetration, reducing the likelihood of off-target neurotoxicity. The compound displays a moderate total clearance rate of 0.987 mL/min/kg, supporting a sustained therapeutic window. Crucially, the model indicates no inherent hepatotoxicity, confirming its compatibility with hepatic tissue. From a transport perspective, Atractylenolide III is not a substrate for P-glycoprotein (P-gp), ensuring it escapes active efflux pump excretion to maintain high intracellular bioavailability. However, it acts as a selective P-gp I inhibitor while remaining inactive against P-gp II. This selective inhibition of P-gp I suggests a valuable synergistic capacity to overcome multidrug resistance (MDR) mechanisms in HCC, potentially sensitizing resistant tumor cells to co-administered chemotherapeutic agents^14^.

**Table 1:** Comprehensive in-silico profiling of physicochemical and ADMET properties.

Parameters	Value	Parameters	Value
Physicochemical Properties*		ESOL Solubility (mg/ml)	4.93E-01
Formula	C15H20O3	ESOL Solubility (mol/l)	1.98E-03
MW (g/mol)	248.32	ESOL Class	Soluble
#Heavy atoms	18.00	Ali Log S	-2.71
#Aromatic heavy atoms	0.00	Ali Solubility (mg/ml)	4.87E-01
Fraction Csp^3^	0.67	Ali Solubility (mol/l)	1.96E-03
#Rotatable bonds	0.00	Ali Class	Soluble
#H-bond acceptors	3.00	Silicos-IT LogSw	-3.15
#H-bond donors	1.00	Silicos-IT Solubility (mg/ml)	1.78E-01
Molar Refractivity	69.15	Silicos-IT Solubility (mol/l)	7.16E-04
TPSA (Å^2^)	46.53	Silicos-IT class	Soluble
Lipophilicity*		Drug-likeness^*^	
iLOGP	2.46	Lipinski #violations	0.00
XLOGP3	2.10	Ghose #violations	0.00
WLOGP	2.70	Veber #violations	0.00
MLOGP	2.87	Egan #violations	0.00
Silicos-IT Log P	3.13	Muegge #violations	0.00
Consensus Log P	2.65	Bioavailability Score	0.55
Pharmacokinetics*		Extra parameters^$#^	
GI absorption	High	Ring Count^#^	3.00
BBB permeant	Yes	QED^#^	0.53
log Kp (cm/s)	-6.32	Toxicity Flags^#^	Non-Toxic
Medicinal Chemistry*		BBB permeability^$^	0.251
PAINS #alerts	0.00	Total Clearance^$^	0.987
Brenk #alerts	1.00	Hepatotoxicity^$^	No
Leadlikeness #violations	1.00	P-glycoprotein substrate^$^	No
Synthetic Accessibility	4.21	P-glycoprotein I inhibitor^$^	Yes
Water Solubility*		P-glycoprotein II inhibitor^$^	No
ESOL Log S	-2.70		

* Calculate using SwissADME, ^#^ RDKit, and ^$^pkCSM.

### Molecular docking analysis

Molecular docking is a key technique in computational drug design (CDD) for identifying potential drug candidates by predicting their binding interactions with target proteins^54^. In this study, 76 protein targets were screened against Atractylenolide III, which was selected based on prior literature and the SwissTargetPrediction database. Proteins were ranked according to their binding affinity and interaction strength, where a higher negative docking score indicates stronger binding and better molecular compatibility with the protein’s active site. The docking results showed that Atractylenolide III exhibited binding affinities ranging from − 9.6 kcal/mol to -5.9 kcal/mol across different target proteins. Among these, the human T-cell surface glycoprotein CD1b displayed the highest binding affinity (-9.6 kcal/mol), while the human crystal structure of CTLA-4 (PDB ID: 1I85) had the weakest interaction (-5.9 kcal/mol) (Table S3). Notably, several classic, established oncogenic drivers highly relevant to HCC pathogenesis and cell proliferation were successfully screened in this 76-protein library^68–71^. These included the serine/threonine-protein kinase AKT (PDB ID: 3O96), which demonstrated a docking affinity of -8.7 kcal/mol; the epidermal growth factor receptor EGFR (PDB ID: 1M17) at -7.9 kcal/mol; the vascular endothelial growth factor receptor VEGFR (PDB ID: 3HNG) at -7.5 kcal/mol; and the tyrosine-protein kinase JAK2 (PDB ID: 4FVQ) at -7.5 kcal/mol. While these classic kinase and receptor targets represent standard pathways of direct intracellular tumor proliferation, CD1b was prioritized for subsequent in silico modeling solely based on its superior thermodynamic docking score (-9.6 kcal/mol), thereby opening an intriguing alternative avenue toward microenvironment-targeted tumor immunomodulation. Consequently, the 1GZP-Atractylenolide III complex was selected for subsequent MD simulations to evaluate binding stability and structural dynamics.

**Fig. 2:**
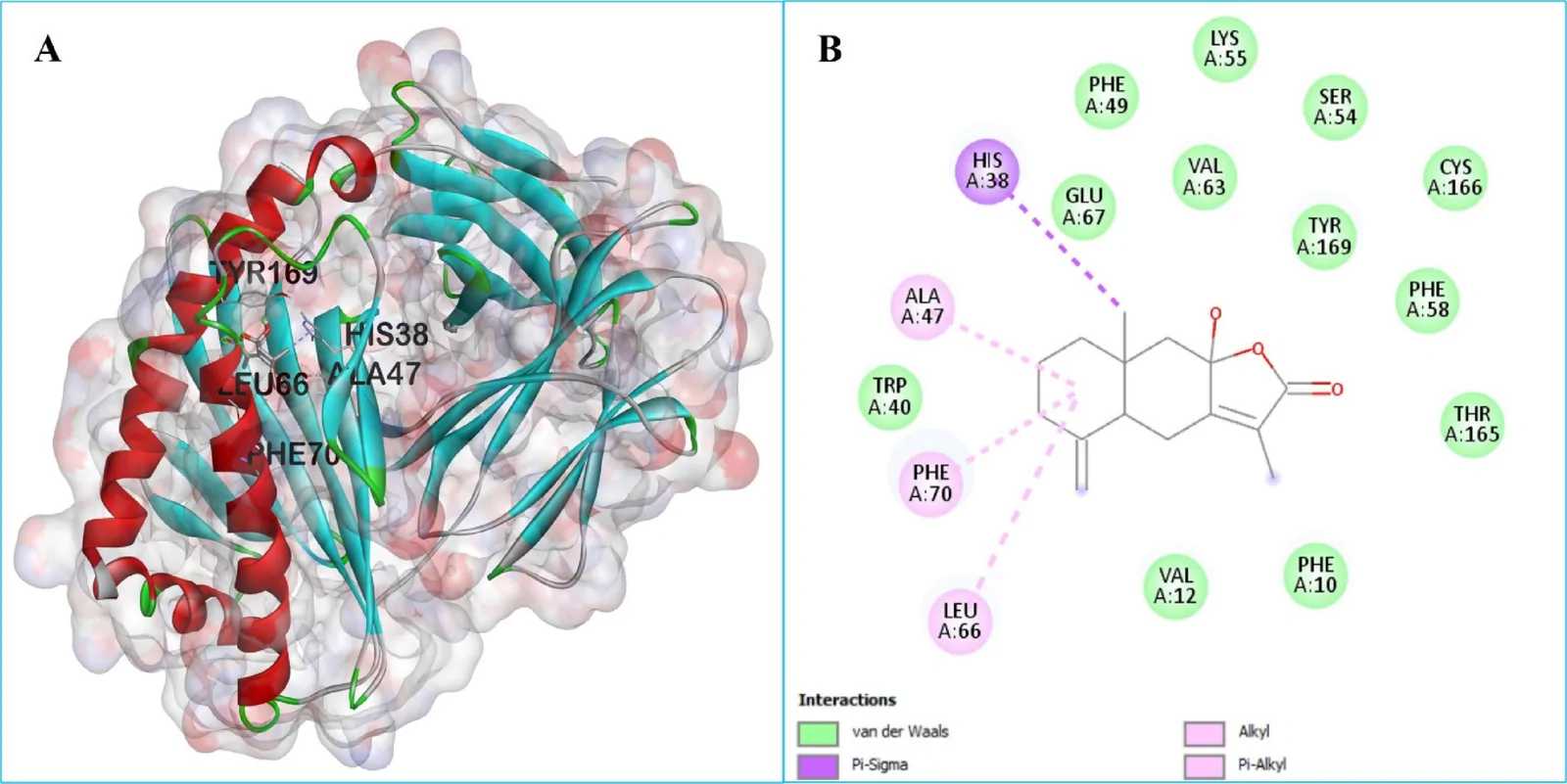
(**A**) A 3D representation showing the surface accessibility area of the interaction between the most stable binding pose of Atractylenolide III and 1GZP. (**B**) A 2D schematic diagram depicting the different types of interactions present in the 1GZP-Atractylenolide III complex.

The docking analysis of 1GZP and Atractylenolide III revealed a strong binding affinity, as illustrated in Fig. 2. Figure 2A depicts the most stable binding pose of Atractylenolide III at the active site of 1GZP, representing the conformation with the highest binding energy. This optimized pose demonstrated multiple interaction types, enhancing its binding stability. The 1GZP-Atractylenolide III docking complex exhibited four primary interaction types: Van der Waals forces, Pi-sigma interactions, alkyl bonds, and Pi-alkyl bonds. Specifically, twelve Van der Waals interactions with VAL12, PHE10, THR165, PHE58, TYR169, CYS166, SER54, VAL63, LYS55, PHE49, GLU67, and TRP40. Also, a single Pi-sigma bond was detected at HIS38. Additionally, two alkyl bonds were found at LEU66 and ALA47. Finally, one Pi-alkyl bond was found at PHE70. These interactions collectively contribute to the potential biological activity of Atractylenolide III within the 1GZP binding site. To ensure the reliability, search-space exhaustiveness, and thermodynamic reproducibility of the docking protocol^72–74^, validation was performed by executing quintuplicate independent docking runs for the top five prioritized receptor-ligand complexes. The binding energy standard deviations across these runs demonstrated exceptional reproducibility, yielding a negligible variance of < 0.05 kcal/mol, with the complete coordinate sets and scoring metrics detailed in Figure S2 and Table S4.

### Role of 1GZP in immune regulation and cancer

1GZP/CD1b is a lipid antigen-presenting molecule that plays a crucial role in immune regulation, particularly in T-cell activation by recognizing bacterial lipid antigens and mediating immune responses against intracellular pathogens^75^. In HCC, exposure to tumor-derived alpha-fetoprotein (AFP) has been shown to significantly reduce the expression of CD1-family molecules on monocyte-derived dendritic cells, thereby weakening natural killer T (NKT) cell function and fostering an immunosuppressive microenvironment^76,77^. The strong binding affinity of Atractylenolide III (-9.6 kcal/mol) with CD1b suggests that it might physically interact with and stabilize the lipid-binding groove of CD1b, potentially rescuing lipid antigen-presentation pathways or preventing its downregulation. It is important to clarify that while direct in vitro cytotoxicity against HepG2 monocultures is primarily mediated by intracellular apoptotic cascades (such as Bax/Bcl-2 modulation)^13^, the predicted interaction with CD1b represents a potiontially highly promising, secondary immunostimulatory mechanism that could act synergistically in vivo. Additionally, 1GZP has been implicated in cancer, particularly lung adenocarcinoma (LUAD), where its expression is associated with improved prognosis and enhanced antitumor immunity^23^. While 1GZP is extensively studied in the context of infectious diseases, such *as Mycobacterium tuberculosis* antigen presentation^78^, its emerging role in oncology highlights a dual therapeutic potential. This underscores the potential of marine-derived bioactive compounds, including Atractylenolide III, as novel candidates for immune-targeted therapies in HCC^14^.

### Molecular dynamics (MD) simulation

**Fig. 3:**
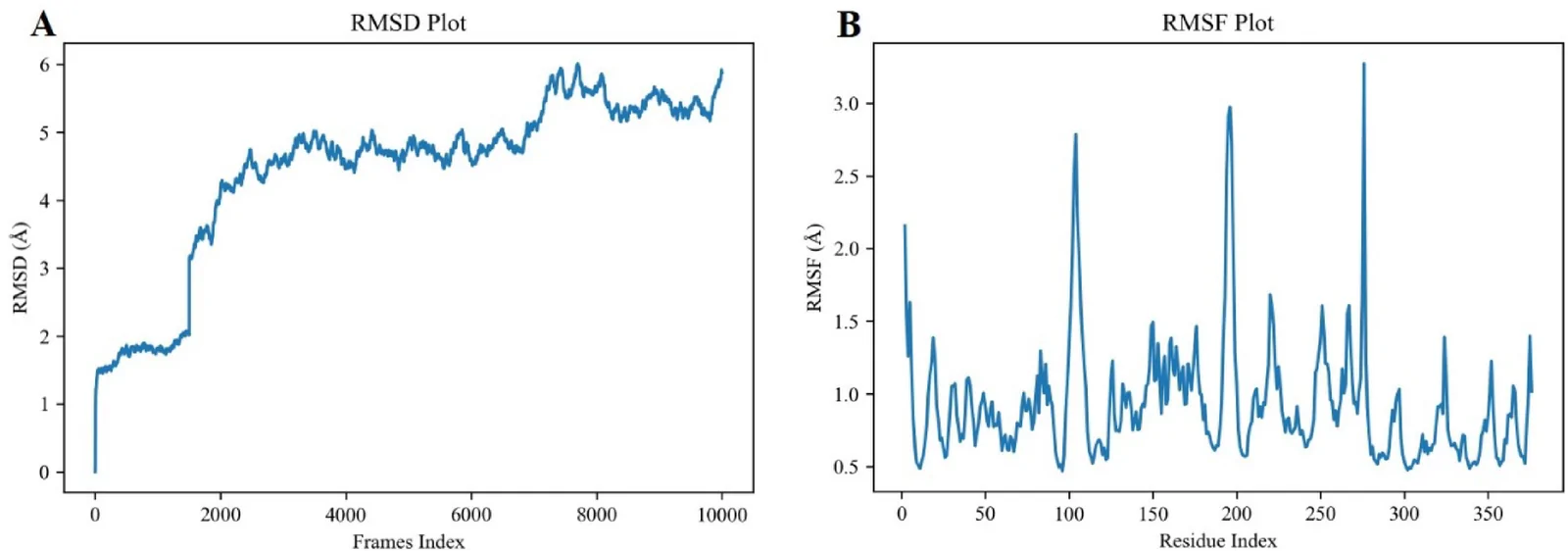
(**A**) RMSD plot illustrating the stability of the 1GZP-Atractylenolide III complex over the simulation period. (**B**) RMSF plot displaying the flexibility of individual amino acid residues within the 1GZP-Atractylenolide III complex.

While molecular docking provides a static snapshot of protein-ligand interactions by assuming a flexible ligand and a rigid receptor, actual biological systems feature intrinsic macromolecular flexibility. To capture these dynamic behaviors under simulated physiological conditions, MD simulations were executed for the 1GZP-Atractylenolide III complex^79^. The structural deviation of the complex over the simulation trajectory was first evaluated using RMSD^80–82^. The complex displayed an average RMSD value of 4.42 Å (Fig. 3A). While an average RMSD of this magnitude indicates a moderate global conformational transition, likely attributed to the flexible outer loops and the expansive, hydrophobic antigen-binding grooves characteristic of the CD1b protein, the trajectory achieved structural equilibrium with minimal subsequent fluctuations, suggesting a relatively thermodynamically stable state was reached. To dissect this behavior at the residue level, the RMSF was analyzed to evaluate local amino acid flexibility^83^. The RMSF profile (Fig. 3B) revealed that key residues comprising the inner binding pocket of 1GZP exhibited restricted fluctuations, demonstrating that the secondary structure and the immediate ligand-binding environment were relatively stable throughout the simulation.

Collectively, the RMSD and RMSF analyses confirm that while the global 1GZP receptor undergoes relative conformational adaptation, the 1GZP-Atractylenolide III pocket interaction maintains structural integrity. Crucially, to address the global structural drift and optimize this binding mode, the stable spatial interactions observed during the simulation were utilized as a spatial blueprint. This structural behavior directly guided our subsequent pharmacophore modeling workflow, which was deployed to screen and identify alternative lead candidates possessing superior structural compatibility, rigid binding pocket fitting, and higher theoretical affinity than Atractylenolide III.

### Pharmacophore modeling and pharmacophore-based virtual screening

Pharmacophore-based virtual screening has become an increasingly valuable approach in drug discovery, particularly when there is no available 3D structural information on the target protein^84^. Even when the 3D structures of targets are accessible, this approach can complement docking-based virtual screening to filter compound libraries effectively. It helps eliminate molecules that lack the essential features necessary for target binding or can serve as a post-filter to refine docking results^19,85^. In the pharmacophore-based virtual screening approach, 3D pharmacophores are generated from known active ligands, target-ligand complexes, or the apo target itself. These pharmacophore models are then screened against virtual libraries of small molecules, identifying compounds that match the defined features required for binding. Compared to traditional in vitro high-throughput screenings, pharmacophore-based virtual screening significantly improves the hit rate and reduces the number of compounds that need to be experimentally tested^84^. While docking-based virtual screening directly simulates the interaction between the ligand and the receptor, pharmacophore-based virtual screening indirectly assesses ligand-target compatibility by evaluating the fit of molecular structures to the pharmacophoric features crucial for binding^86^. Consequently, the pharmacophore-based approach effectively identifies and ranks potential hits without considering the actual interaction dynamics between ligands and targets^19^.

As previously described, each pharmacophore model was designed based on the most stable binding pose of the ligand. The developed pharmacophore hypothesis included two hydrophobic regions, one hydrogen bond donor, and two hydrogen bond acceptors. Additionally, specific criteria were set, including an RMSD value below 0.35 Å, a molecular weight of ≤ 500 g/mol, and a maximum of 10 rotatable bonds. Following this, the pharmacophoric features were used to screen the ZINC purchasable database, resulting in a collection of 1513 compounds. Subsequently, a pharmacophore-based virtual screening was conducted using AutoDock Vina. The primary goal of virtual screening is to filter a compound library to obtain a subset of molecules with a high likelihood of exhibiting the desired biological activity. The efficiency of virtual screening can be assessed by its ability to enrich compounds with favorable binding affinities. According to Table S5, 315 compounds demonstrated a higher binding affinity than the isolated compound. Among them, ZINC64701878 exhibited the most significant binding affinity, achieving a score of -12.7 kcal/mol, highlighting its potential as a strong candidate for further investigation.

Additionally, the top-ranking compounds from the virtual screening were further analyzed using RDKit. As shown in Table S5, ZINC64701878 successfully meets Lipinski’s Rule of Five criteria, which suggests favorable drug-likeness. Specifically, it has a molecular weight of 487.53 g/mol (≤ 500 g/mol), 3 hydrogen bond donors (≤ 5), 4 hydrogen bond acceptors (≤ 10), and a LogP value of 1.93 (≤ 5)^56^. Additionally, the compound’s 8 rotatable bonds (≤ 10) and a topological polar surface area (TPSA) of 90.63 Å² (≤ 140 Å²) align with Veber’s criteria, indicating potential for good oral bioavailability and permeability^57^. These characteristics imply that ZINC64701878 may exhibit a favorable ADMET profile, contributing to its enhanced drug-likeness and potential as a lead compound.

**Fig. 4:**
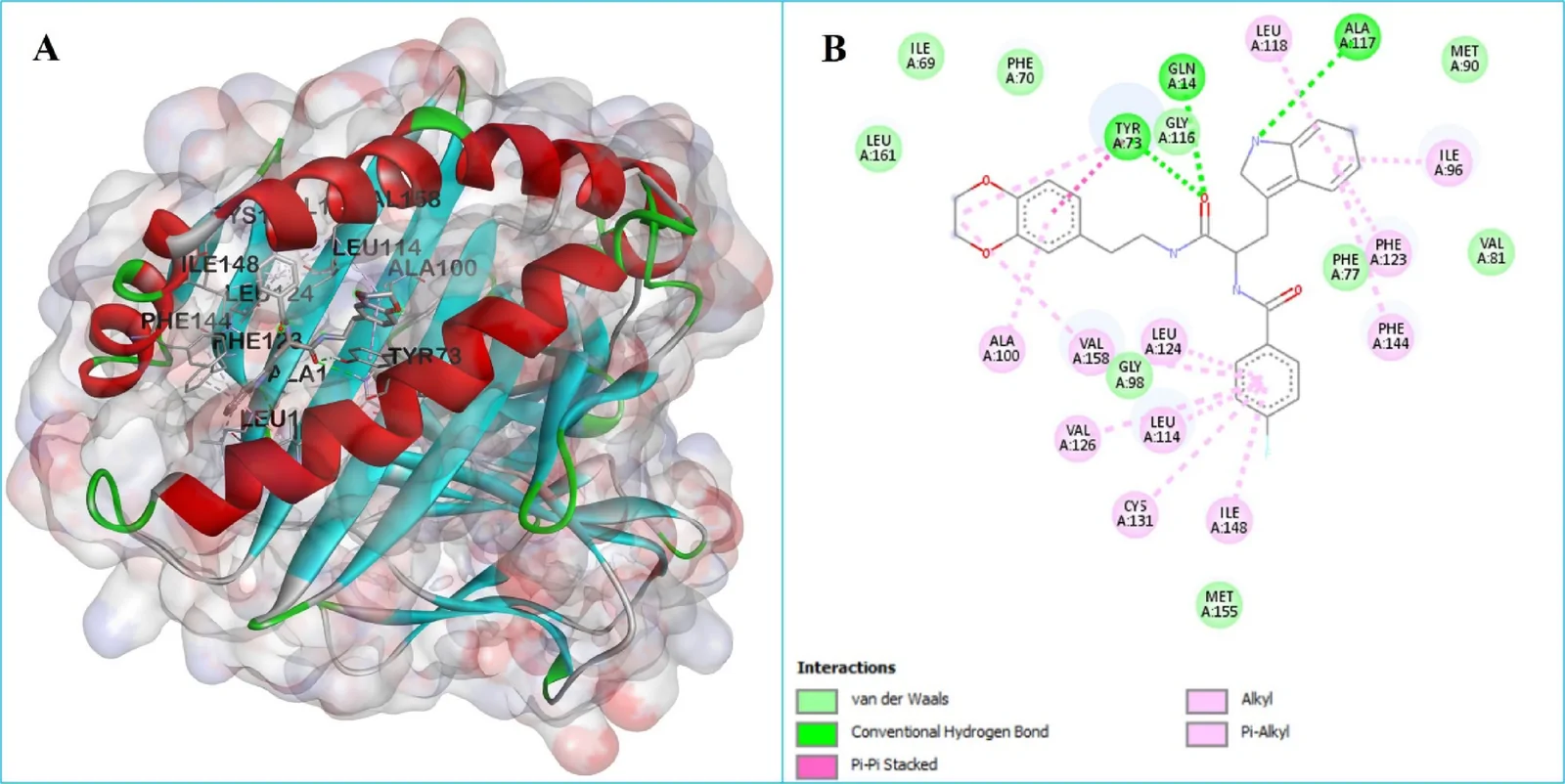
(A) 3D representation of the surface accessibility interaction between 1GZP and ZINC64701878. (B) 2D schematic diagram highlighting the different types of interactions present in the 1GZP-ZINC64701878 complex.

Moreover, the docking analysis between 1GZP and ZINC64701878 showed a robust binding interaction (Fig. 4A,B). The docking complex featured five distinct types of interactions: Van der Waals forces, hydrogen bonds, Pi-sigma interactions, alkyl bonds, and Pi-alkyl bonds. Specifically, eight Van der Waals interactions were identified with MET155, GLY98, VAL81, PHE77, MET90, GLY116, LEU161, ILE69, and PHE70. Additionally, three conventional hydrogen bonds were formed with GLN14, TYR73, and ALA117. A single Pi-Pi stacked interaction was observed at TYR73. The complex also exhibited three alkyl bonds with ILE96, LEU118, and VAL158. Notably, ten Pi-alkyl interactions occurred with TYR73, PHE123, PHE144, LEU114, LEU124, VAL126, CYS131, ILE148, VAL158, and ALA100 (Fig. 4B). These diverse interaction types likely contribute to the stability and strong binding affinity observed in the docking study, suggesting a significant potential for ZINC64701878 as a promising lead compound.

### Comprehensive quantum molecular descriptor and MEP analysis

Table 2 summarizes the quantum molecular descriptors obtained from the DFT calculations for Atractylenolide III and ZINC64701878. These descriptors offer valuable insights into the electronic properties, reactivity, and interaction capabilities of the molecules. To gain deeper insights into the electronic structures, reactivity patterns, and chemical stability of the prioritized compounds before future synthetic cycles, Density Functional Theory (DFT) evaluations were conducted. Frontier molecular orbitals (FMOs) play a definitive role in determining both the chemical reactivity and the kinetic stability of a molecular system^87^. Within this framework, the energy of the Highest Occupied Molecular Orbital (E_HOMO_) quantifies the capacity of a molecule to donate electrons, where higher HOMO values indicate a stronger nucleophilic character^88^. Conversely, the energy of the Lowest Unoccupied Molecular Orbital (E_LUMO_) reflects the ability of a molecule to accept electrons, with lower LUMO energies designating a highly effective electrophile^89^.

Firstly, the frontier molecular orbital band gap energy (ΔE), which represents the discrete energy difference between the HOMO and LUMO layers (E_HOMO_ - E_LUMO_), serves as a primary indicator of a molecule’s electronic excitation potential and overall kinetic stability^90^. The significantly smaller band gap (ΔE) of ZINC64701878 (1.86 eV) compared to Atractylenolide III (4.04 eV) indicates lower kinetic stability and higher chemical reactivity. This narrow gap facilitates easier intra- and intermolecular charge-transfer interactions with the active site residues of the targeted receptor. These results collectively suggest a higher electronic reactivity and a greater propensity to participate in dynamic charge-transfer processes critical for biological recognition, directly correlating with the superior binding affinity observed for ZINC64701878 (Table 2)^20^. Secondly, global chemical reactivity descriptors, specifically chemical hardness (η) and softness (S), are intrinsically interrelated with this energy gap and serve to quantify a molecule’s relative resistance to charge transfer^91^. Chemical hardness and softness are inversely related, meaning softer molecules possess a more polarizable electron cloud and are generally more chemically reactive. ZINC64701878 demonstrates lower hardness and higher softness, indicating an increased chemical reactivity and polarizability that allows its electronic structure to adjust effectively within fluctuating macromolecular environments^92,93^. These traits are essential in dynamic biological environments, where molecular flexibility and electronic adaptability enhance target interaction potential^20^. Concurrently, the baseline stability of these structures correlates with their respective hardness parameters, where high chemical hardness reflects resistance to electronic deformation that predicts a highly favorable, stable behavior during subsequent synthetic processing^94^.

Thirdly, electronegativity (χ) and electrophilicity (ω) were calculated to critically assess electron-attracting behaviors and the thermodynamic driving forces governing target engagement. Electronegativity dictates the spatial delocalization of electrons across the entire molecular framework^91^. ZINC64701878 displayed elevated electronegativity and a remarkably high electrophilicity index. Because the electrophilicity index indicates the overall directional flow of electrons between a donor and an acceptor, a higher electrophilicity value underscores a strongly electrophilic nature and an enhanced capacity to accept electrons^95^. Crucially, a high electrophilicity index directly translates to a superior binding affinity with organic target molecules^96^, suggesting a powerful potential to engage in robust, stable interactions with electron-rich biological sites that may enhance downstream therapeutic efficacy^20^.

**Fig. 5:**
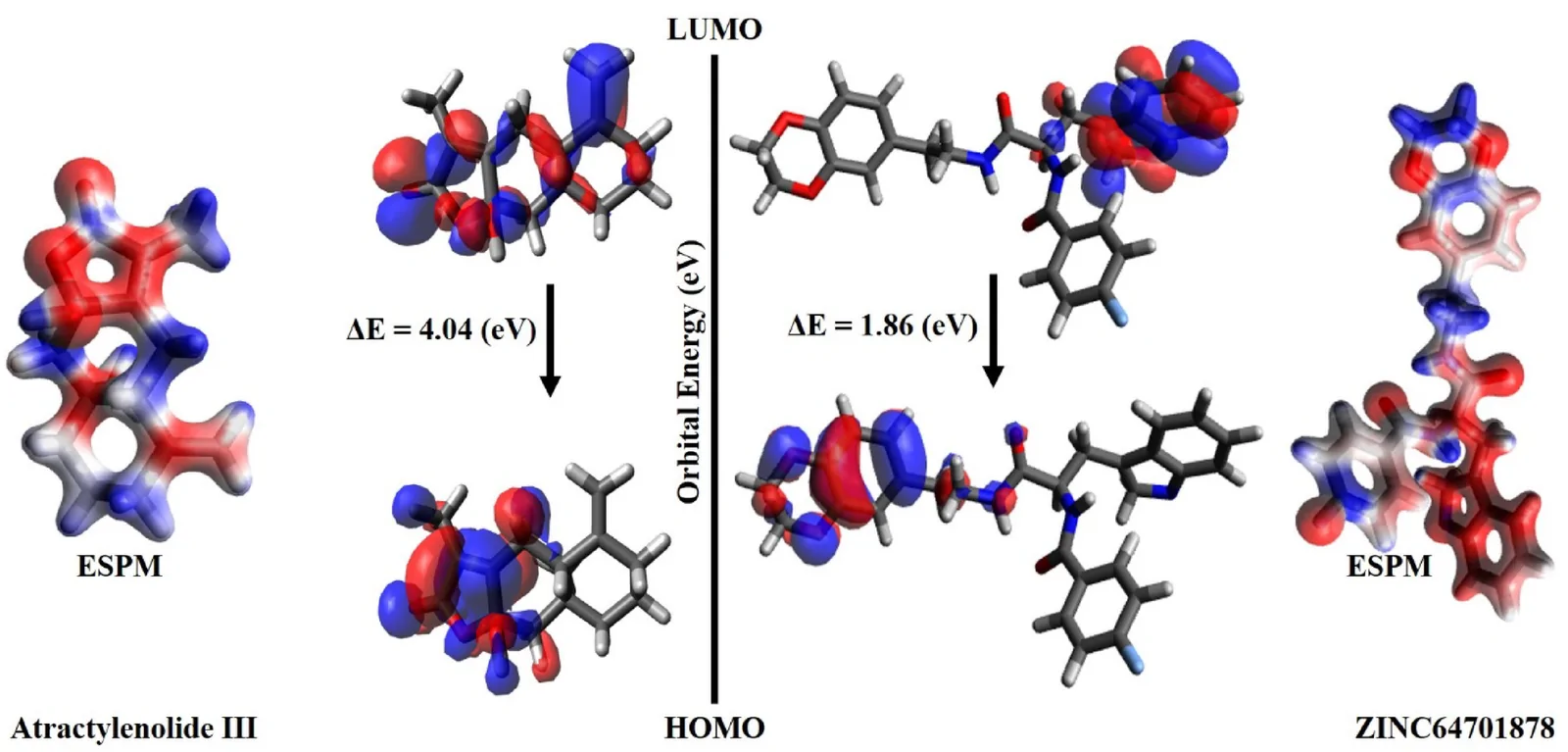
Density functional theory (DFT) analysis of Atractylenolide III and ZINC64701878, including the electrostatic potential map (ESPM).

Lastly, Molecular Electrostatic Potential (MEP) analysis (Fig. 5) was utilized to map the electrophilic and nucleophilic regions of the molecules. While MEP analysis primarily describes electrostatic charge distributions rather than directly proving binding affinity, the distinct charge localization on ZINC64701878 helps visualize the electrostatic complementary features that likely contribute to its interaction with the receptor’s binding pocket. Overall, the integration of DFT and MEP analyses provided a comprehensive evaluation of the electronic and reactivity profiles of the compounds. The reduced band gap, increased softness, and high electrophilicity of ZINC64701878 indicate not only theoretical promise but also potential effectiveness in real-world biological applications.

**Table 2:** Results of DFT calculations for Atractylenolide III and ZINC64701878.

ID	E_HOMO_ (eV)	E_LUMO_ (eV)	Band Gap (ΔE)	Hardness (η)	Softness (λ)	Electronegativity (χ)	Electrophilicity (ω)
Atractylenolide III	-6.04	-2.01	4.04	2.02	0.50	4.03	4.01
ZINC64701878	-5.01	-3.14	1.86	0.93	1.07	4.07	8.89

## Conclusion

This study highlights the cytotoxic potential of Atractylenolide III, a compound isolated from the marine coral *Tubipora musica*, against liver cancer (HepG2 cells). Computational insights revealed its strong binding affinity (-9.6 kcal/mol) with 1GZP, validated through molecular docking and molecular dynamics (MD) simulations. Further pharmacophore modeling identified ZINC64701878, a derivative with even higher binding affinity (-12.7 kcal/mol) and favorable pharmacokinetic properties, outperforming Atractylenolide III in reactivity and stability as confirmed by density functional theory (DFT). These findings underscore the promise of Atractylenolide III and its similarities as lead compounds for liver cancer treatment, particularly given the urgent need for natural, low-toxicity agents to address the global burden of HCC, which disproportionately affects males. The integration of experimental isolation with computational drug design, including docking, dynamics, pharmacophore modeling, and DFT, proved critical in elucidating molecular interactions and optimizing therapeutic potential. However, this study is limited by several factors: First, the 1 ns molecular dynamics simulation timeframe served strictly as an initial structural viability filter; future studies should employ longer, microsecond-scale simulations to explore more extensive thermodynamic and global conformational changes. Second, while in vitro cytotoxicity was verified in HepG2 cell lines, genetic target-validation studies (such as site-directed mutagenesis or CD1b knockout assays) are required to definitively isolate the biological contribution of CD1b to the compound’s overall pharmacological profile. Third, the lack of standard chemotherapeutic controls and selectivity profiles on normal cell lines must be addressed in future phases. Consequently, future work should prioritize in vivo studies to confirm systemic bioactivity, evaluate safety margins on normal hepatocytes, and synthesize prioritized virtual screening analogs like ZINC64701878 for direct experimental validation. Ultimately, this research advances the paradigm of marine-derived anticancer agents and demonstrates the power of combining natural product discovery with computational tools to accelerate drug development against challenging diseases like HCC.

## Supplementary Information

Below is the link to the electronic supplementary material.

Supplementary Material 1

Supplementary Material 2

## Data Availability

The data that support the findings of this study are available upon reasonable request from the corresponding author.
