# Supplementary material for: Integrated experimental and computational evaluation of atractylenolide III from Tubipora musica for anticancer lead identification
Source: Sci Rep. 2026 Sep 9;16:28208. doi: 10.1038/s41598-026-68577-5 (PMC13558756; doi:10.1038/s41598-026-68577-5)
Supplement: Supplementary file 1 [file 41598_2026_68577_MOESM2_ESM.docx]

**Supporting Information**

**Integrated Experimental and Computational Evaluation of Atractylenolide III from *Tubipora musica* for Anticancer Lead Identification**

Hamada A. A. Noreldeen*

National Institute of Oceanography and Fisheries, NIOF, Cairo, Egypt.

*Corresponding author, Hamada A. A. Noreldeen ([haali8651@gmail.com](mailto:haali8651@gmail.com)).

**Figure S1.** NMR characterization of the isolated natural product: (A) ^1^H-NMR spectrum and (B) ^13^C-NMR spectrum of Atractylenolid III in CDCl_3_

**Figure S2.** Three-dimensional crystal structures of the top 5 prioritized receptor proteins.

**Table S1:**^1^H NMR spectral data of Atractylenolid III in CDCl_3_.

| Position | *δ_H_ (J in Hz)* |
| --- | --- |
| 1 | 1.23 m |
| 2 | 1.63 m |
| 3a | 2.35 d (13.02) |
| 3b | 1.96 m |
| 4 | ………… |
| 5 | 1.83 s |
| 6a | 2.42 t (12.36) |
| 6b | 2.6 d (13.08) |
| 7 | …………. |
| 8 | …………. |
| 9a | 1.55 t (26.82) |
| 9b | 2.24 d (13.74) |
| 10 | ………… |
| 11 | ………….. |
| 12 | …………. |
| 13 | 1.80 s |
| 14 | 1.01 s |
| 15a | 4.58 s |
| 15b | 4.85 s |

(s=singlet, d=duplet, t=triplet, q=quartet, b=broad, m= multiplet)

**Table S2:**^13^C NMR spectral data of Atractylenolid III in CDCl_3_.

| Position | *δ*C |
| --- | --- |
| 1 | 41.4 t |
| 2 | 22.4 t |
| 3 | 36.2 t |
| 4 | 148.9 s |
| 5 | 51.8 d |
| 6 | 24.6 t |
| 7 | 160 .8 s |
| 8 | 103.4 s |
| 9 | 51.4 t |
| 10 | 36.7 s |
| 11 | 122.3 s |
| 12 | 172.0 s |
| 13 | 8.3 s |
| 14 | 16.6 s |
| 15 | 106.8 t |

Multiplicity was determined by DEPT experiments (s=quaternary, d=methane, t=methylene, q=methyl)

**Table S3a.** Binding affinity scores of the top 76 protein targets against Atractylenolide III with an exhaustiveness level of 8.

| **Receptor** | **Best Binding Affinity (kcal/mol)** | | **RMSD l.b.** | | **RMSD u.b.** | **Time Taken (seconds)** |
| --- | --- | --- | --- | --- | --- | --- |
| **1gzp** | -9.6 | 0 | | 0 | | 11.36 |
| **1onq** | -9.4 | 0 | | 0 | | 14.66 |
| **5i6x** | -9.4 | 0 | | 0 | | 10.58 |
| **1pob** | -9.3 | 0 | | 0 | | 9.88 |
| **4m48** | -9.2 | 0 | | 0 | | 12.22 |
| **1zt4** | -9.1 | 0 | | 0 | | 13.58 |
| **1gv4** | -9 | 0 | | 0 | | 20.77 |
| **1sqn** | -9 | 0 | | 0 | | 11.74 |
| **2am9** | -8.8 | 0 | | 0 | | 11.29 |
| **4qtb** | -8.8 | 0 | | 0 | | 11.05 |
| **3o96** | -8.7 | 0 | | 0 | | 13.57 |
| **5hk1** | -8.7 | 0 | | 0 | | 12.83 |
| **3elj** | -8.6 | 0 | | 0 | | 12.66 |
| **3lkj** | -8.6 | 0 | | 0 | | 13.44 |
| **2rd0** | -8.5 | 0 | | 0 | | 15.36 |
| **1djx** | -8.3 | 0 | | 0 | | 12.48 |
| **1kvo** | -8.3 | 0 | | 0 | | 10.9 |
| **1q3w** | -8.3 | 0 | | 0 | | 10.67 |
| **5kir** | -8.3 | 0 | | 0 | | 15.49 |
| **6u9w** | -8.2 | 0 | | 0 | | 11.87 |
| **1akj** | -8.1 | 0 | | 0 | | 13.27 |
| **1xjd** | -8.1 | 0 | | 0 | | 11.02 |
| **3jus** | -8.1 | 0 | | 0 | | 13.38 |
| **3mop** | -8.1 | 0 | | 0 | | 15.1 |
| **4dqy** | -8.1 | 0 | | 0 | | 10.73 |
| **1hck** | -8 | 0 | | 0 | | 11.02 |
| **3eqm** | -8 | 0 | | 0 | | 12.8 |
| **3fxi** | -8 | 0 | | 0 | | 11.93 |
| **3k6s** | -8 | 0 | | 0 | | 10.83 |
| **1m17** | -7.9 | 0 | | 0 | | 11.56 |
| **1n45** | -7.9 | 0 | | 0 | | 10.5 |
| **1u59** | -7.9 | 0 | | 0 | | 10.57 |
| **2p5e** | -7.9 | 0 | | 0 | | 10.66 |
| **1l6j** | -7.8 | 0 | | 0 | | 11.63 |
| **5hi3** | -7.8 | 0 | | 0 | | 12.98 |
| **1jxq** | -7.7 | 0 | | 0 | | 13.49 |
| **2j0j** | -7.7 | 0 | | 0 | | 13.39 |
| **1ouk** | -7.6 | 0 | | 0 | | 12.2 |
| **1yjd** | -7.6 | 0 | | 0 | | 12.23 |
| **3hng** | -7.5 | 0 | | 0 | | 12.35 |
| **4fvq** | -7.5 | 0 | | 0 | | 9.86 |
| **4l4t** | -7.5 | 0 | | 0 | | 12.18 |
| **1m48** | -7.4 | 0 | | 0 | | 8.7 |
| **1tup** | -7.4 | 0 | | 0 | | 12.44 |
| **1ck7** | -7.3 | 0 | | 0 | | 14.39 |
| **1gfw** | -7.3 | 0 | | 0 | | 9.79 |
| **2az5** | -7.3 | 0 | | 0 | | 12.11 |
| **2b5i** | -7.3 | 0 | | 0 | | 13.27 |
| **4hg7** | -7.3 | 0 | | 0 | | 9.63 |
| **1tcr** | -7.2 | 0 | | 0 | | 12.61 |
| **2imt** | -7.2 | 0 | | 0 | | 10.7 |
| **2r79** | -7.2 | 0 | | 0 | | 11.73 |
| **4jsv** | -7.2 | 0 | | 0 | | 12.09 |
| **1fos** | -7.1 | 0 | | 0 | | 9.94 |
| **1itb** | -7.1 | 0 | | 0 | | 11.85 |
| **1maz** | -7.1 | 0 | | 0 | | 10.24 |
| **1qb0** | -7.1 | 0 | | 0 | | 10.94 |
| **1qtn** | -7.1 | 0 | | 0 | | 9.47 |
| **1g5m** | -7 | 0 | | 0 | | 10.17 |
| **4u6w** | -7 | 0 | | 0 | | 11.24 |
| **1a02** | -6.9 | 0 | | 0 | | 11.34 |
| **1alu** | -6.9 | 0 | | 0 | | 11.74 |
| **1f16** | -6.9 | 0 | | 0 | | 10.72 |
| **1bg1** | -6.8 | 0 | | 0 | | 11.73 |
| **1nfi** | -6.7 | 0 | | 0 | | 11.2 |
| **2b9r** | -6.7 | 0 | | 0 | | 12.49 |
| **1cwr** | -6.6 | 0 | | 0 | | 11.02 |
| **1pty** | -6.6 | 0 | | 0 | | 10.08 |
| **1sy6** | -6.6 | 0 | | 0 | | 12.47 |
| **6dvw** | -6.6 | 0 | | 0 | | 12.43 |
| **3eto** | -6.5 | 0 | | 0 | | 9.26 |
| **1c25** | -6.2 | 0 | | 0 | | 10.8 |
| **1wio** | -6.2 | 0 | | 0 | | 7.7 |
| **3rrq** | -6 | 0 | | 0 | | 10.13 |
| **1hrc** | -5.9 | 0 | | 0 | | 9.49 |
| **1i85** | -5.9 | 0 | | 0 | | 11.11 |

**Table S3b.** Complete list of evaluated receptor PDB IDs and their corresponding formal biological names**.**

| PDB ID | Full Name |
| --- | --- |
| 1gzp | CD1b |
| 1onq | CD1a |
| 5i6x | Serotonin Transporter (SERT) |
| 1pob | Phospholipase A2 group 1B (PLA2G1B) |
| 4m48 | Dopamine Transporter (DAT) (homology model) |
| 1zt4 | CD1d |
| 1gv4 | Apoptosis-Inducing Factor (AIF) |
| 1sqn | Progesterone Receptor (PGR) |
| 2am9 | Androgen Receptor (AR) |
| 4qtb | Extracellular Signal-Regulated Kinases 1 and 2 (ERK1/2) |
| 3o96 | AKT |
| 5hk1 | Sigma Opioid Receptor (SIGMAR1) |
| 3elj | C-Jun-N-terminal Protein Kinase 1/2 (JNK1/2) |
| 3lkj | CD40L (CD40 Ligand) |
| 2rd0 | PI3K (Phosphoinositide 3-Kinase) |
| 1djx | Phosphatidylinositol-Specific Phospholipase C (PLCg1) |
| 1kvo | Phospholipase A2 group IIA (PLA2G2A) |
| 1q3w | GSK-3beta (Glycogen Synthase Kinase 3 Beta) |
| 5kir | Cyclooxygenase-2 (COX-2) |
| 6u9w | P2X Purinoceptor 7 (P2RX7) |
| 1akj | CD8 |
| 1xjd | Protein Kinase C (PKC) |
| 3jus | Cytochrome P450 51 (CYP51) (homolog) |
| 3mop | MyD88 |
| 4dqy | Poly-(ADP)-Ribose Polymerase (PARP) / PARP1 |
| 1hck | CDK1 (Cyclin-Dependent Kinase 1) |
| 3eqm | Cytochrome P450 19A1 (CYP19A1) |
| 3fxi | TLR4 (Toll-Like Receptor 4) |
| 3k6s | Leukocyte Function-Associated Antigen (LFA-1) |
| 1m17 | EGFR (Epidermal Growth Factor Receptor) |
| 1n45 | Heme Oxygenase 1 (HMOX1) (homolog) |
| 1u59 | Zeta-Associated Protein of 70 Kd (ZAP-70) |
| 2p5e | dCTP Pyrophosphatase 1 (DCTPP1) |
| 1l6j | MMP-9 (Matrix Metalloproteinase-9) |
| 5hi3 | IL-17A (Interleukin 17A) |
| 1jxq | Casp-9 (Caspase 9) |
| 2j0j | Protein Tyrosine Kinase 2 Beta (PTK2B) |
| 1ouk | p38 Mitogen-Activated Protein Kinase (p38 MAPK) |
| 1yjd | CD28 |
| 3hng | VEGFR (Vascular Endothelial Growth Factor Receptor) |
| 4fvq | JAK2 |
| 4l4t | MR1 (MHC-Related Protein 1) |
| 1m48 | IL-2 (Interleukin 2) |
| 1tup | p53 |
| 1ck7 | MMP-2 (Matrix Metalloproteinase-2) |
| 1gfw | Casp-3 (Caspase 3) |
| 2az5 | TNF-alpha (Tumor Necrosis Factor Alpha) |
| 2b5i | IL-2R (CD25) |
| 4hg7 | p53-Binding Protein Mdm-2 (MDM2) |
| 1tcr | T-cell Receptor (TCR) |
| 2imt | Bak (Bcl-2 homologous antagonist/killer) |
| 2r79 | Tubulin--tyrosine ligase (TTL) |
| 4jsv | Molecular Target of Rapamycin (mTOR) |
| 1fos | Activator Protein 1 (AP-1) |
| 1itb | IL-1beta (Interleukin 1 Beta) |
| 1maz | Bcl-XL (B-cell lymphoma-extra large) |
| 1qb0 | Dual specificity phosphatase Cdc25B (CDC25B) |
| 1qtn | Casp-8 (Caspase 8) |
| 1g5m | Bcl-2 (B-cell lymphoma 2) |
| 4u6w | CD1c |
| 1a02 | Nuclear Factor of Activated T-cells (NFAT) |
| 1alu | IL-6 (Interleukin 6) |
| 1f16 | Bax (Bcl-2-associated X protein) |
| 1bg1 | STAT3 |
| 1nfi | Nuclear Factor-kB (NF-kB) |
| 2b9r | Cyclin B1 |
| 1cwr | Cdc25c |
| 1pty | Protein-tyrosine phosphatase 1B (PTP1B) |
| 1sy6 | CD3 |
| 6dvw | Transient Receptor Potential Cation Channel Subfamily V Member 3 (TRPV3) |
| 3eto | Notch |
| 1c25 | Dual specificity phosphatase Cdc25A (CDC25A) |
| 1wio | CD4 |
| 3rrq | Programmed Death-1 (PD-1) |
| 1hrc | Cytochrome c |
| 1i85 | CTLA-4 (Cytotoxic T-Lymphocyte-Associated Protein 4) |

**Table S4.** Re-docking validation parameters for the top 5 receptors against Atractylenolide III (mean binding energy ± SD based on n = 5 independent replicates) alongside exhaustive grid box spatial coordinates and RMSD values. The table additionally includes the parallel re-docking validation parameters and structural metrics for receptor 1GZP against the virtual screening lead compound ZINC64701878 (n = 5, mean ± SD). (exhaustiveness = 8)

| Receptor | Ligand | X | Y | Z | Size | Avarage | RMSD l.b. | RMSD u.b. |
| --- | --- | --- | --- | --- | --- | --- | --- | --- |
| 1gzp | Atractylenolide III | 58.07 | 18.60 | -2.35 | 45 | -9.52 ± 0.04 | 0 | 0 |
|  | ZINC64701878 | 67.30 | 15.65 | -3.09 | 49 | -12.66 ± 0.04 | 0 | 0 |
| 1onq | Atractylenolide III | 18.42 | 0.50 | 52.64 | 40 | -9.40 ± 0.00 | 0 | 0 |
| 5i6x | Atractylenolide III | -44.08 | -10.56 | 0.84 | 33 | -9.40 ± 0.00 | 0 | 0 |
| 1pob | Atractylenolide III | 10.59 | 13.81 | 36.87 | 40 | -9.30 ± 0.00 | 0 | 0 |
| 4m48 | Atractylenolide III | -39.06 | -1.82 | 55.22 | 34 | -9.20 ± 0.00 | 0 | 0 |

**Table S5.** Docking parameters and drug-likeness and physicochemical properties of the top 315 compounds against 1GZP at an exhaustiveness level of 8 (provided in a separate Excel sheet).
